# Author Correction: Genome-wide real-time in vivo transcriptional dynamics during *Plasmodium falciparum* blood-stage development

**DOI:** 10.1038/s41467-022-29067-0

**Published:** 2022-03-15

**Authors:** Heather J. Painter, Neo Christopher Chung, Aswathy Sebastian, Istvan Albert, John D. Storey, Manuel Llinás

**Affiliations:** 1 Department of Biochemistry and Molecular Biology, The Pennsylvania State University, University Park, PA 16802 USA; 2 Huck Center for Malaria Research, The Pennsylvania State University, University Park, PA 16802 USA; 3 Lewis-Sigler Institute for Integrative Genomics and Department of Molecular Biology, Princeton University, Princeton, NJ 08544 USA; 4 Huck Institutes of the Life Sciences, The Pennsylvania State University, University Park, PA 16802 USA; 5 Center for Statistics and Machine Learning, Princeton University, Princeton, NJ 08544 USA; 6 Department of Chemistry, The Pennsylvania State University, University Park, PA 16802 USA; 7 Present Address: Institute of Informatics, Faculty of Mathematics, Informatics, and Mechanics, University of Warsaw, 02-097 Warsaw, Poland

Correction to: *Nature Communications* 10.1038/s41467-018-04966-3, published online 9 July 2018.

The original version of the Supplementary Information associated with this Article included an incorrect Supplementary Data 1 file, in which data for ‘30 hpi’ was duplicated in ‘29 hpi’ for ‘Total Abundance’ and ‘Total Estimated Abundance’ of genes. The HTML has been updated to include a corrected version of Supplementary Data 1.

## Supplementary information

Corrected Supplementary Data 1

