# Supplementary material for: Genome-wide real-time in vivo transcriptional dynamics during Plasmodium falciparum blood-stage development
Source: Nat Commun. 2018 Jul 9;9:2656. doi: 10.1038/s41467-018-04966-3 (PMC6037754; doi:10.1038/s41467-018-04966-3)
Supplement: Supplementary file 1 — Supplementary Information [file 41467_2018_4966_MOESM1_ESM.pdf]

Supplementary Information to:

Genome-wide Real-time *in vivo* Transcriptional Dynamics During *Plasmodium falciparum* Blood-stage Development

Painter *et al.*

Supplementary Figure 1:

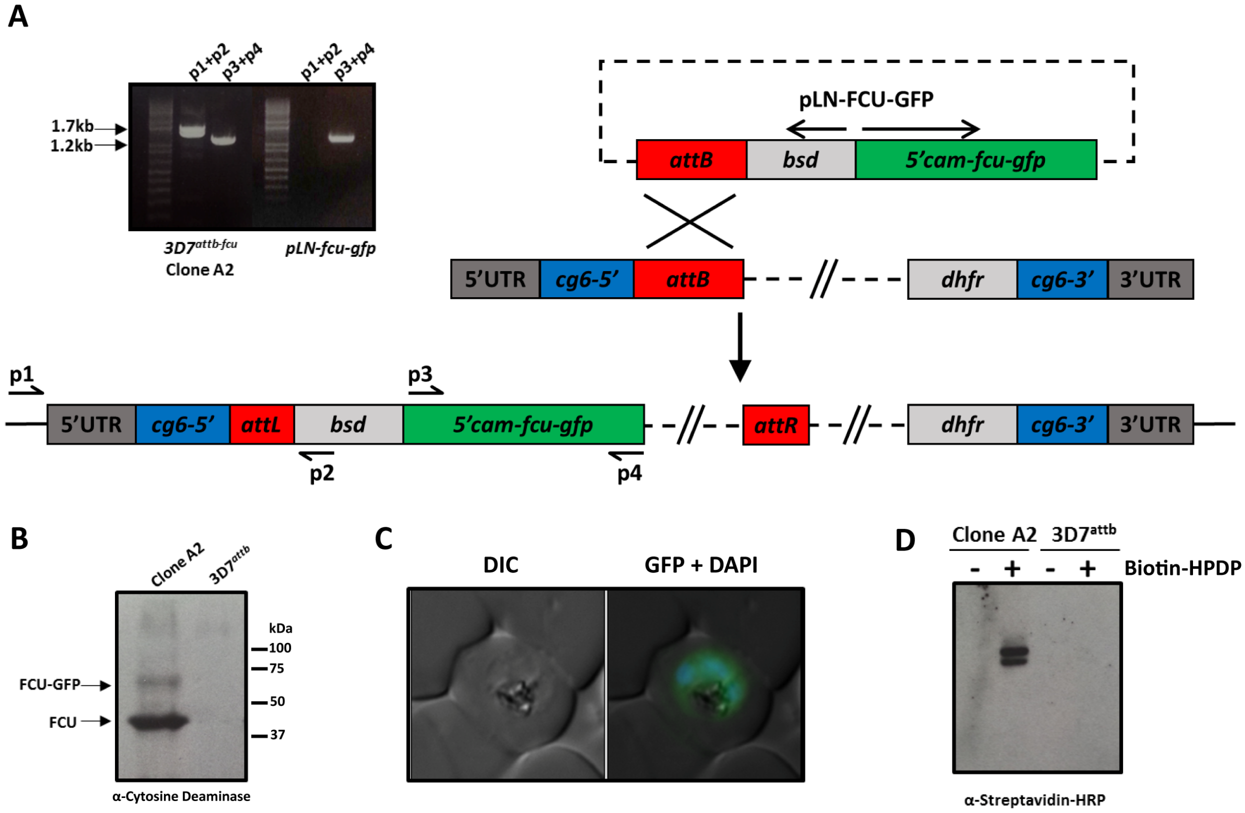

Supplementary Figure 1: Generation of *P. falciparum* capable of pyrimidine salvage via *fcu-gfp* genome integration. A) Schematic for generation of *P. falciparum* transgenic strain 3D7$^{attb::FCU-GFP}$ via integrase-mediated *attB* x *attP* recombination as described previously (*73*). Integration (p1 + p2) and presence (p3 +p4) of the *fcu-gfp* fusion gene. **B)** Western blot verification of FCU-GFP expression in 3D7$^{attb::FCU-GFP}$ clone A2 parasites versus wild-type parasites, 3D7^{attb} probed with anti-yeast cytosine deaminase antibody (1:250). Expression of FCU-GFP in 3D7$^{attb::FCU-GFP}$ parasites was verified by **C)** Fluorescence microscopy (GFP = green, nuclear DNA stained with DAPI = blue) and **D)** Northern blot detecting incorporation of 4-TU into parasite ribosomal RNA. Both wild-type 3D7^{attb} and 3D7$^{attb::FCU-GFP}$ parasites were grown for 12 hours in the presence of 4-TU (40µM). The specificity of RNA thiol-incorporation and biotinylation was assessed by running 2µg of each RNA sample with and without EZ-link Biotin-HPDP incubation. Total RNA was transferred to a nylon membrane and probed with streptavidin-HRP to detect biotinylated RNAs.

Supplementary Figure 2:

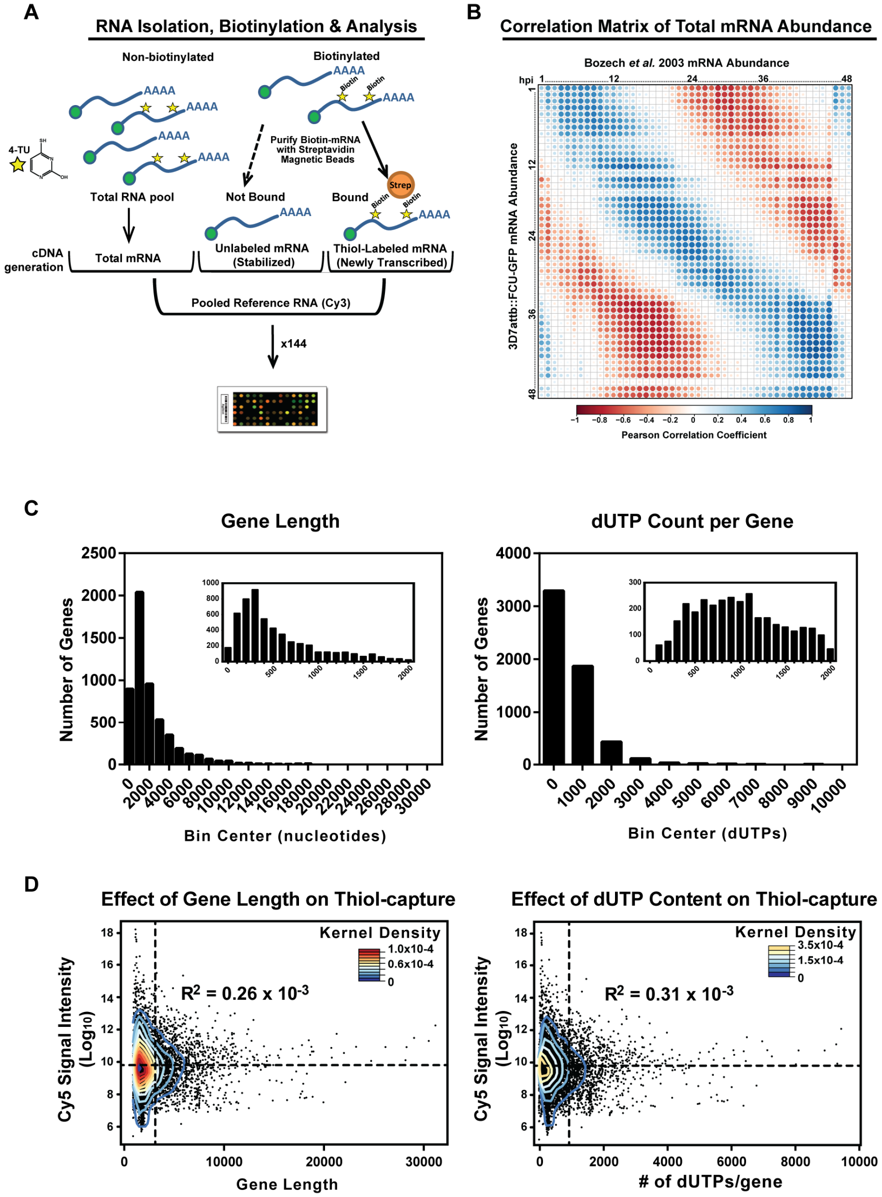

Supplementary Figure 2: Capture of mRNA dynamics during the IDC of *P. falciparum*. A) Following a 10 min pulse with 40 µM 4-TU, total RNA is extracted. Nascent thiolated-RNA can be biotinylated and affinity purified by streptavidin magnetic beads for analysis by cDNA microarray. Labeled thiol-RNA represents transcript generated during the 10min pulse with 4-TU, while the unlabeled mRNA represents transcripts that existed prior to the 4-TU pulse and were stabilized or not turned-over. **B)** Hourly correlation of total mRNA abundance captured in this experiment to a previously published transcriptome (median Pearson *corr* = 0.72) **C)** Histogram representation of transcript lengths and dUTP content genome-wide with insets representing expanded binning of 0-2000. **D)** Correlation plot of the captured labeled transcripts measured by cDNA microarray (Cy5 signal intensity at 24hpi) versus both their gene length and dUTP content.

Supplementary Figure 3:

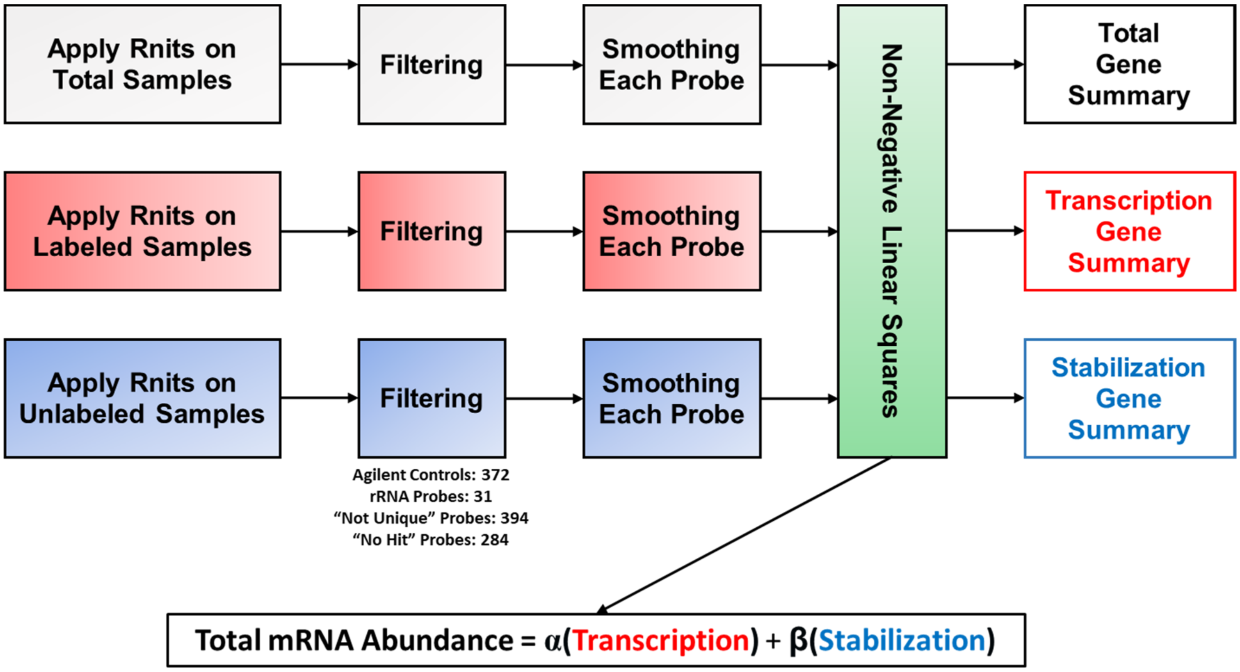

Supplementary Figure 3: Schematic of the normalization and modeling method. To quantitatively determine the contribution of nascent transcription and stabilization to the total abundance profiles of each gene, the three timecourses (Total, Labeled, and Unlabeled) were separately normalized by Rnits. After normalization, probes classified as Agilent Controls, rRNAs, "Not Unique" or "No Hit" probes were filtered from the data-set. The profile of each probe was smoothed across the 48 h timecourse. The contribution of transcription and stabilization to the total mRNA abundance (see equation) for each gene was estimated by Non-Negative Least Squares (NNLS) coefficients, α and β. Resulting calculated expression values for each probe were then averaged across the representative gene using Tukey's Biweight.

Supplementary Figure 4:

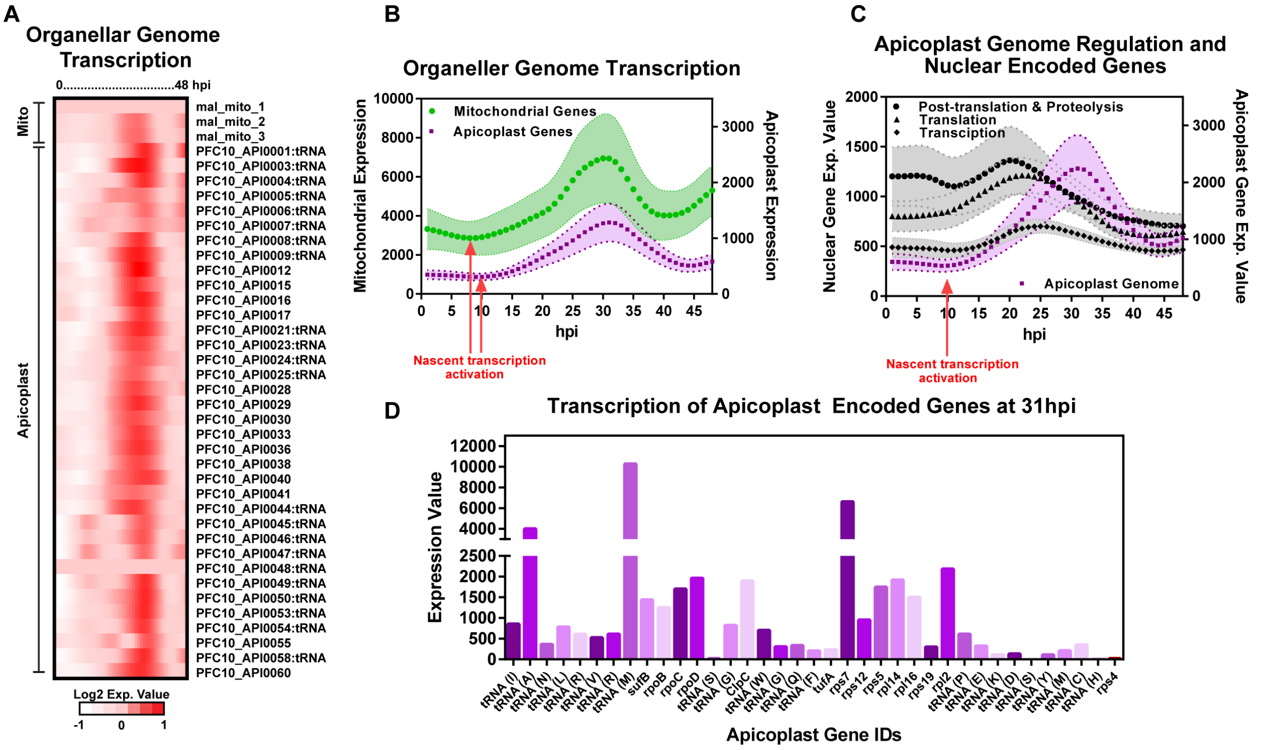

Supplementary Figure 4: Capture of organellar genome transcription. A) Heatmap representation of normalized and centered nascent transcription expression values from both the mitochondrial and apicoplast genomes. **B)** Mean expression values of both the mitochondrial (green) and apicoplast (purple) genome encoded transcripts (± s.e.m). Red arrow indicates the timing of nascent transcription. **C)** Mean expression values of apicoplast genome (purple) encoded transcripts compared to nuclear genome (black) encoded transcripts involved in transcription and expression of apicoplast genes (± s.e.m). Red arrow indicates the timing of nascent transcription. **D)** Calculated nascent transcript expression values of apicoplast encoded genes at the peak time of transcription (31hpi). Genes are ordered by arrangement on the apicoplast genome.

Supplementary Figure 5:

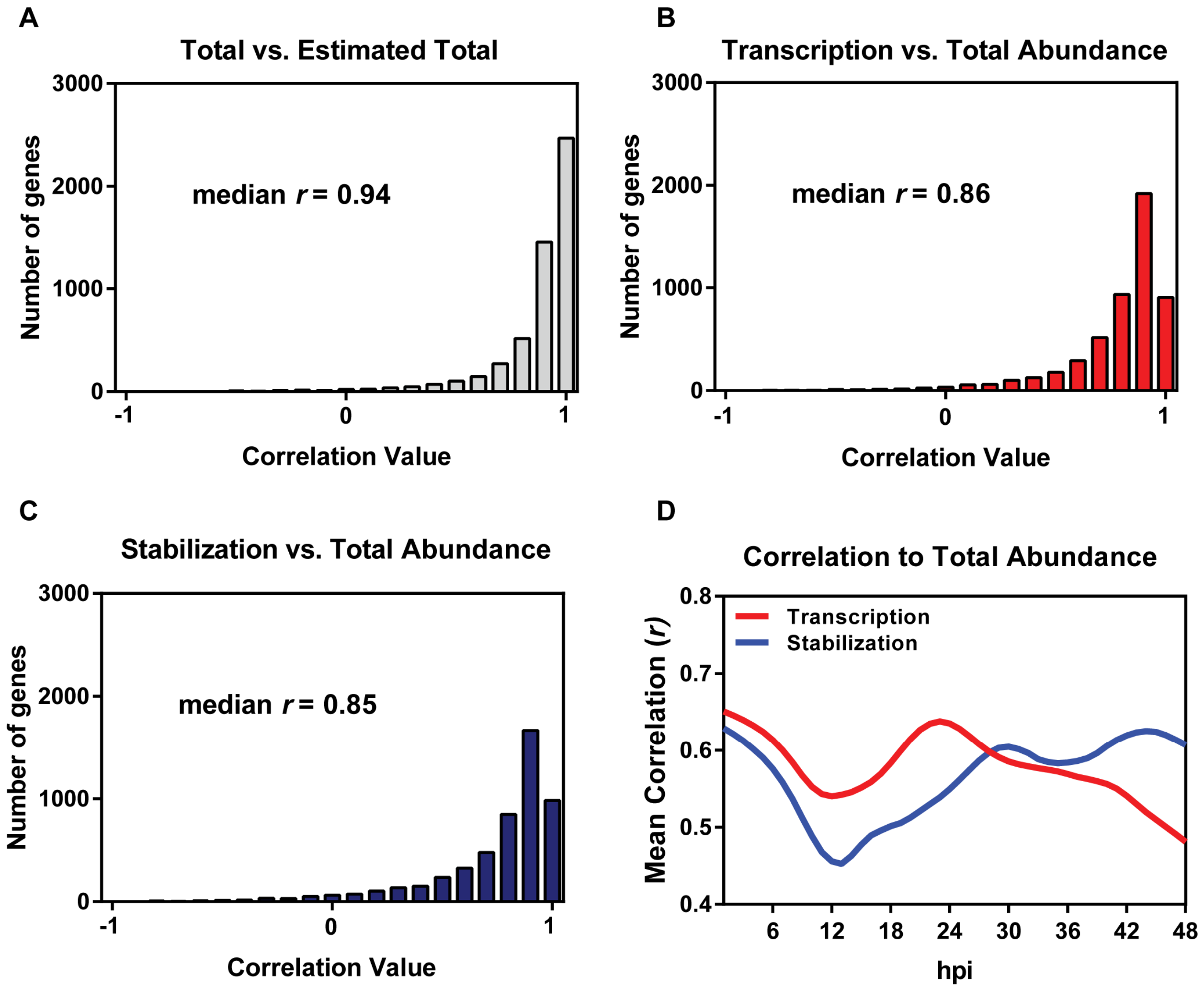

Supplementary Figure 5: Gene-to-gene correlation of mRNA dynamics to total abundance. Histogram representation of correlation of gene expression values throughout the IDC captured by cDNA microarray between A) total mRNA abundance and the modeled estimated total abundance; B) nascent transcription and total mRNA abundance; and C) stabilization and total mRNA abundance. D) Gene-to-gene mean correlation (*r*) values of both transcription (▬) and stabilization (▬) mean correlation to mRNA total abundance were plotted throughout the IDC.

Supplementary Figure 6:

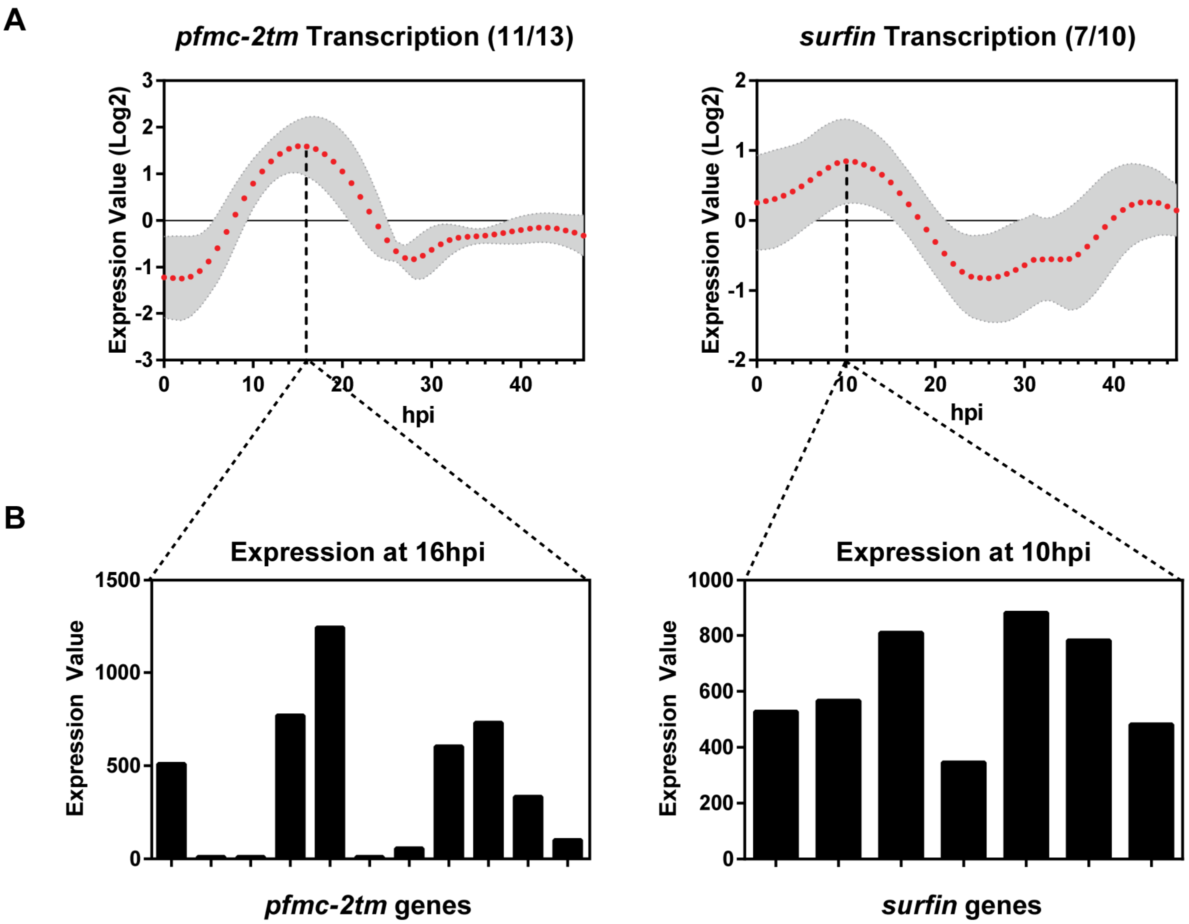

Supplementary Figure 6: Additional variant surface antigen gene families. A) Transcription of surface expressed gene families; *pfmc-2tm* (11/13), *surfins* (7/10) are displayed as the mean (log2 Expression Value) and ± s.e.m. throughout the 48h IDC. The hpi where peak transcription of each gene family is displayed as a dropline in each graph. **B)** Bar graph representation of the expression value for each *pfmc-2tm* and *surfin* gene at the peak time of transcription.

Supplementary Figure 7:

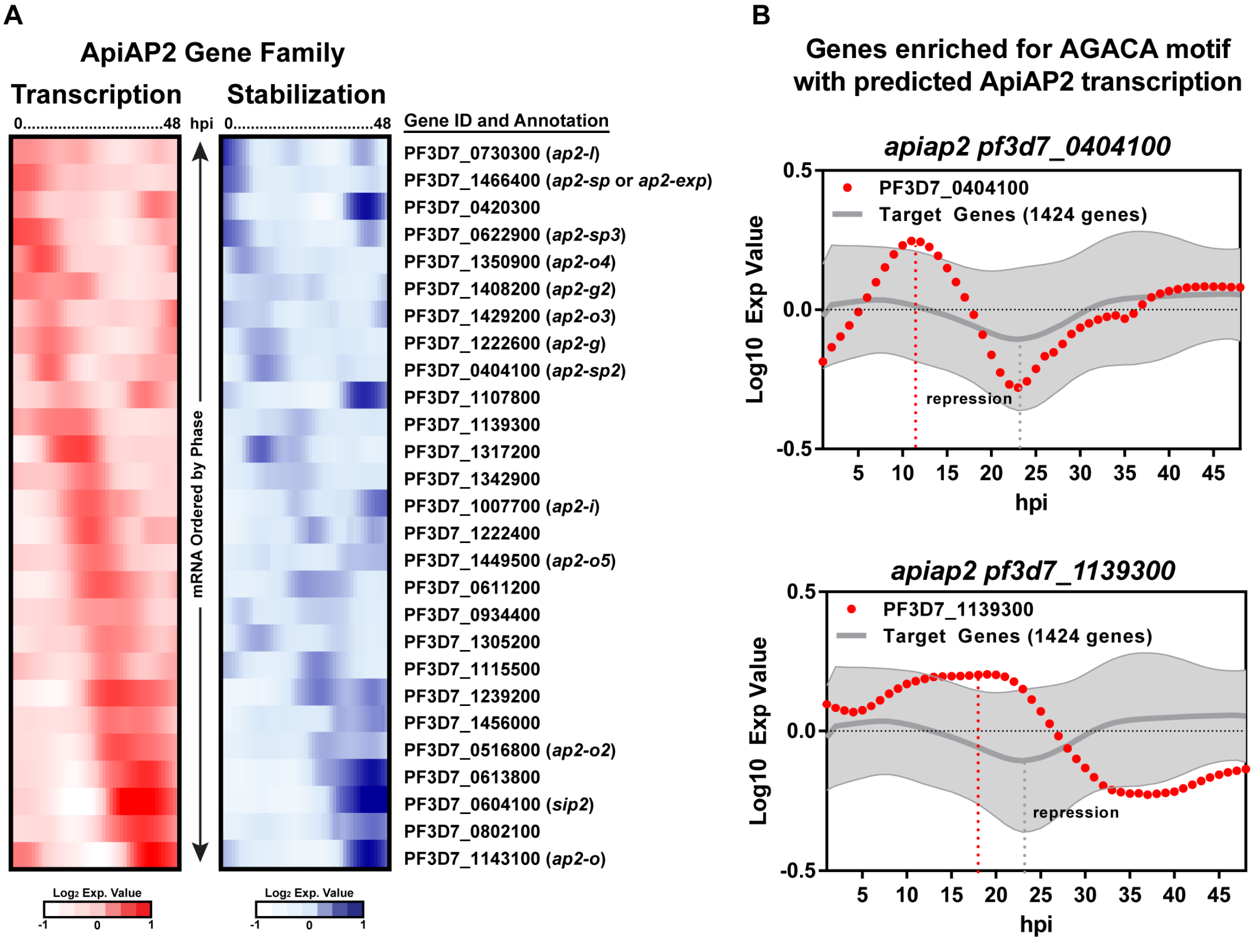

Supplementary Figure 7: Predicting the function of *trans*-acting factors from profiles of nascent transcription. **A)** The nascent transcription or stabilization expression values representing all members ApiAP2 gene family. The expression values were normalized, centered and ordered based on peak timing of transcription. **B)** The nascent transcription profile of genes identified by FIRE to be enriched with the AGACA motif are plotted (grey, mean Log10 Expression Value and s.e.m.) in comparison to the transcription profiles of potential *Pf*ApiAP2 *trans*-acting factors (red), *pf3D7_0404100* (top panel) and *pf3D7_1139300* (bottom panel).
