# Supplementary material for: Genome-wide real-time in vivo transcriptional dynamics during Plasmodium falciparum blood-stage development
Source: Nat Commun. 2018 Jul 9;9:2656. doi: 10.1038/s41467-018-04966-3 (PMC6037754; doi:10.1038/s41467-018-04966-3)
Supplement: Supplementary file 3 — Description of Additional Supplementary Files [file 41467_2018_4966_MOESM3_ESM.pdf]

Description of Additional Supplementary Files

Supplementary Data 1: Genome-wide Calculated Expression Values and Peak Timing for 48h IDC Timecourse. Summary of normalized and calculated expression values for each timecourse where individual tabs of the spreadsheet represent the Total Abundance, Transcription, Stabilization, and Estimated Total Abundance (based on the summation of both Transcription and Stabilization). Within tabs, each gene and annotation is listed (Columns A-B), along with hourly expression values (Columns C-AX), and timing of peak expression with *p*-value significance calculated by the Lomb-Scargle Periodogram approach (Columns AY-AZ).

Supplementary Data 2: Summary of Calculated Rates of Transcription and Decay. Rates of transcription and decay for each gene (Column A) ordered by the stage of development at which the peak timing of transcription or stabilization occurs (Column B). Rate is represented as the number of transcripts (peak expression value) nascently transcribed or decayed in one hour (Column C).

Supplementary Data 3: Summary of GO-Term Enrichments. The genome-wide peak timings of transcription and stabilization for each gene were binned into 30 minute intervals throughout the IDC and displayed as a histogram (Fig. 3). Intervals of peaks in transcription and stabilization were distributed throughout the IDC with distinct 'bursts' and 'troughs'. Genes contained within each 'burst' were analyzed for GO-term enrichment using the analysis tool on http://www.PlasmoDB.org.
